# Adaptation of the transcriptional response to resistance exercise over 4 weeks of daily training

**DOI:** 10.1096/fj.202201418R

**Published:** 2022-12-05

**Authors:** Mark R. Viggars, Hazel Sutherland, Hermann Lanmüller, Martin Schmoll, Manfred Bijak, Jonathan C. Jarvis

**Affiliations:** ^1^ Research Institute for Sport & Exercise Sciences, Liverpool John Moores University Liverpool UK; ^2^ Department of Physiology and Aging University of Florida Gainesville Florida USA; ^3^ Myology Institute, University of Florida Gainesville Florida USA; ^4^ Center for Medical Physics and Biomedical Engineering Medical University of Vienna Vienna Austria

## Abstract

We present the time course of change in the muscle transcriptome 1 h after the last exercise bout of a daily resistance training program lasting 2, 10, 20, or 30 days. Daily exercise in rat tibialis anterior muscles (5 sets of 10 repetitions over 20 min) induced progressive muscle growth that approached a new stable state after 30 days. The acute transcriptional response changed along with progressive adaptation of the muscle phenotype. For example, expression of type 2B myosin was silenced. Time courses recently synthesized from human exercise studies do not demonstrate so clearly the interplay between the acute exercise response and the longer‐term consequences of repeated exercise. We highlight classes of transcripts and transcription factors whose expression increases during the growth phase and declines again as the muscle adapts to a new daily pattern of activity and reduces its rate of growth. *Myc* appears to play a central role.

**Abbreviations**
Amd1: Adenosylmethionine decarboxylase 1
Ankrd1: Ankyrin repeat domain 1
AP‐1: activator protein 1
Arntl: brain and muscle Arnt like factor 1
ATF: activating transcription factor
Bgn: Biglycan
bHLH: basic helix–loop–helix
Clock: Circadian locomoter output cycles protein kaput
Col1a1: Collagen Type I alpha 1 chain
Col3a1: Collagen Type III alpha 1 chain
CPN: common peroneal nerve
Creb: cAMP response element‐binding protein
CSA: cross‐sectional area
DEG: differentially expressed gene
EDL: extensor digitorum longus
Ehd: EH domain containing 1
Fos: Fos proto‐oncogene, AP‐1 transcription factor subunit
Hmox1: Heme oxygenase 1
IPG: implantable pulse generator
Jund: JunD proto‐oncogene, AP‐1 transcription factor subunit
Lox: Lysyl oxidase
Max: MYC‐associated factor X
Mef2c: myocyte enhancer factor 2C
MoTrPAC: Molecular Transducers of Physical Activity Consortium
Mt‐atp: mitochondrial encoded ATP synthase subunit
Mt‐co: mitochondrial encoded cytochrome c oxidase
Mt‐nd: mitochondrial encoded NADH: Ubiquinone Oxidoreductase core subunits
Myc: MYC, basic helix loop helix transcription factor
Myh: Myosin heavy chain
Nfatc3: nuclear factor of activated T cells 3
Npas2: neuronal PAS domain protein 2
PCA: principal component analysis
Phkg1: phosphorylase b kinase
Pygm: glycogen phosphorylase
SOM: Self Organizing Map
TA: tibialis anterior
Tcf3: transcription factor 3
USF1: upstream transcription factor 1

## 1 INTRODUCTION

Intermittent resistance exercise training of skeletal muscle provides mechanical and metabolic overload that induces hypertrophy and increases maximum force.^1^, ^2^ Maintained muscle strength reduces the risk of metabolic diseases and premature mortality and facilitates independent living in older life.^3^, ^4^, ^5^ Muscle hypertrophy requires changes in gene expression that increase cell growth and translational capacity and suppress negative regulators of those processes.^1^, ^2^ Chronic overload by synergist muscle ablation,^6^, ^7^, ^8^ electrical stimulation under repeated anesthesia,^9^, ^10^ or conditioned squat like exercises^11^ have been used to investigate the hypertrophic response. However, they differ from typical human resistance training comprising sets of intermittent loaded contractions.^12^ Instead, they induce a continuous static load, or training distributed in time (especially if effort is linked to a food reward), or the repeated stress of anesthesia. The NIH‐funded Molecular Transducers of Physical Activity Consortium (MoTrPAC) has not so far included a pre‐clinical animal model of resistance exercise training toward their goal of mapping the molecular responses to exercise. We present data from a new model based on programmed co‐contraction of lower limb muscles in rats. We refer to “SpillOver” exercise in text and figures because activation of the ankle dorsiflexors by electrical stimulation of the entire common peroneal nerve is adjusted to spill over to the adjacent tibial nerve to provide controlled resistance to those dorsiflexors by partial antagonistic activation of the plantarflexors.

Microarray and RNA‐sequencing technologies have uncovered the most influential or most differentially expressed gene transcripts (DEGs) across acute and chronic training datasets, sex, and length of training in humans.^13^, ^14^, ^15^, ^16^, ^17^, ^18^, ^19^, ^20^ However, no study has assessed the time course of changes in the transcriptome as muscle adapts to an ongoing hypertrophic stimulus. Recent studies inferred some aspects of the time course of changes in the acute transcriptomic response to resistance exercise in blood and muscle^21^ through meta‐analyses of acute and long‐term responses. Here, we characterize the precise time course of hypertrophic adaptation to repeated acute resistance exercise as muscle is trained and accommodates to the resistance exercise stimulus, in the sense that the acute response is reduced, and compare our data with those inferred time courses. Two studies show the time course of gene expression during hypertrophy after synergist ablation, but they suggest a process of overload, degeneration, and repair rather than adaptation.^7^, ^8^

There are complex interactions between the time course of the acute response to an exercise bout over a few hours, and the longer‐term responses that occur over days or weeks of repeated exercise. We measured progressive changes over 30 days in the acute transcriptional response 1‐h after a 20‐min resistance training session delivered once per day. Such daily exercise produces hypertrophy of the TA muscle, that slows after approximately 20 days. After that time even though daily resistance training is continued, there is no further muscle growth. We presume, then, that the cellular processes for hypertrophy decline because of changes in the muscle that reduce the internal stimulus for growth despite ongoing daily exercise.

We used RNA‐sequencing to characterize the genes, pathways, and networks associated with this time course of muscle hypertrophy. We hypothesize that genes or gene families whose response to an acute exercise session is up or downregulated in the early sessions of training, but then returns to near control levels over the 30‐day time course will include important regulators for inducing or suppressing muscle growth. Network analysis identified the basic helix–loop–helix domain (bHLH) transcription factor (TF) *Myc*, as a master regulator of the transcriptional regulation of hypertrophy. Its expression in response to acute resistance exercise declines as the muscle becomes adapted to a new pattern of daily training and stops growing.

## 2 MATERIALS AND METHODS

### 2.1 Experimental design

The animal experiments were conducted under the provisions of the Animals (Scientific Procedures) Act 1986 and approved by the British Home Office (PA6930221). Forty‐seven male Wistar rats were group‐housed with 2–3 per cage maintaining an alternating 12 h light 12 h dark cycle. The mean age when euthanized was 23 ± 2 weeks. Mean pre‐ and post‐body masses were as follows for sham surgery (376 ± 33 g to 390 ± 36 g, *n* = 5), 2 days of training (487 ± 72 g to 480 ± 69 g, *n* = 7), 10 days of training (412 ± 66 g to 423 ± 59 g, *n* = 12), 20 days of training (433 ± 101 g to 472 ± 89 g, *n* = 10), and 30 days of training (447 ± 74 g to 503 ± 74 g, *n* = 13). No significant differences were present between groups pre‐training. The sham surgery group had significantly lower body mass than the 30‐day training group sham group post‐training (*p* = .046), likely attributed to their shorter experimental timecourse and therefore reduced body fat accumulation.

### 2.2 Resistance training protocol & pattern

Animals received 1 session per day of SpillOver resistance training in the left hind‐limb via stimulation from an implanted pulse generator (IPG) as previously described,^22^ for 2, 10, 20, or 30 days or underwent sham surgery. Training was conducted within the first 2 h of the relatively inactive light cycle of the rat. Briefly, for high load (SpillOver) exercise to elicit slight stretch under load, the dorsiflexor muscles, tibialis anterior (TA), and extensor digitorum longus (EDL), received supramaximal activation via a cathode placed underneath the common peroneal nerve (CPN), while the anode was positioned underneath the tibial nerve and the stimulation current was adjusted if necessary by remote programming, to recruit enough of the gastrocnemius, plantaris, and soleus (plantarflexor muscles) to provide resistance against the contraction of the dorsiflexors.

After the initial programming, daily training was delivered automatically by the IPG and consisted of an initial 10 seconds of preparatory stimulation at a low frequency (F = 4 Hz, phase width = 258 μs, current = approximately 1 mA), followed by 5 sets of 10 tetanic contractions at 100 Hz. Each contraction lasted for 2 s with 2 s rest between contractions and 2.5 min of rest between sets. The stimulation was delivered only in the left hind‐limb, so muscles of the right hind‐limb act as unstimulated contralateral controls. Stimulation with these settings and the amplitude chosen to balance dorsiflexion and plantarflexion described above was well‐tolerated by all animals without further anesthesia or sedation. Regular observations during daily training across the time course revealed no adverse behavioral signs.

### 2.3 Electrical stimulation

Silicone encapsulated radio frequency controlled implantable pulse generators (IPGs) (MiniVStim 12B, Competence Team for Implanted Devices, Center for Medical Physics and Biomedical Engineering, Medical University Vienna, Austria) were used to deliver impulses. These IPG's are an advanced development of the previously described 12A version^23^ offering additional functionality crucial for this study. With approximately cylindrical dimensions of 20 mm length and 16 mm diameter, the implant was well tolerated by the rats, as known from previous studies.^22^, ^24^ In contrast to the previous 12A version, the 12B version was controlled wirelessly via an external programming device and an Android driven tablet computer (Xperia Tablet Z, Sony Corporation, Tokyo, Japan). This allowed for fine adjustment (8‐bit resolution) of the stimulation amplitude individually for each animal. After initial setup, the customized stimulation pattern was transferred to the implant, enabling automatic daily delivery of SpillOver stimulation to the targeted nerves. To optimize the temporal accuracy of the autonomously operating IPG's, the internal clock‐frequency of the microprocessor was measured by the control software and used to adjust the register values of the stimulation patterns to compensate for temporal deviations. This process was performed during every transfer of a stimulation pattern and enabled a temporal accuracy of approximately ±1% (i.e., delivering stimulation every 24 h would yield to a deviation of less than ±15 min between two training sessions). The electronic circuit was connected to a lithium 1/3 N battery (170mAh), thus offering an expected lifetime of ~ more than 6 months with the daily stimulation pattern noted above and a typical pulse amplitude of 1.2 mA.

### 2.4 Surgical procedure

Animals were anesthetized during implant procedures by inhalation of a gaseous mixture of isoflurane in oxygen at approximately 3% for induction and 1%–2% for maintenance. Once anesthetized, a subcutaneous injection of Enrofloxacin (5 mg/kg‐1 body mass) (Baytril®) and an intramuscular injection of Buprenorphine (0.05 mg/kg‐1 body mass) (Temgesic, Indivior, Slough, UK) into the right quadriceps were administered with strict asepsis maintained throughout the procedure. The IPG's were implanted into the abdominal cavity accessed by a lateral incision through the skin and peritoneum, between the rib cage and pelvis on the left side of the animal. A polyester mesh attached to the IPG was incorporated into the suture line closing the peritoneum, securing the device against the abdominal wall. Two PVC‐insulated stainless‐steel electrode leads (Cooner Sales Company, Chatsworth, California, USA) with terminal conductive loops, were fed through the peritoneal incision and tunneled under the skin to the lateral side of the upper left hind‐limb. A second incision was made through the skin and biceps femoris muscle to give access to the CPN under which the cathode was placed (to stimulate the dorsiflexors). The anode was placed in the muscular tissue deep to the tibial nerve about 5 mm distal to its bifurcation from the sciatic nerve to allow SpillOver stimulation to produce additional partial activation of the plantarflexors to resist the contraction of the dorsiflexors. All incisions were closed in layers and 7 days were allowed for recovery from surgery before the start of the training protocol.

### 2.5 Muscle sampling, preservation, and RNA isolation

Animals were euthanized using rising concentrations of carbon dioxide, followed by cervical dislocation 1 h post their last exercise bout. TA muscles from both hind limbs were immediately harvested, cleaned of excess connective tissue, and weighed. The mid‐belly of the TA was cut out, placed on cork for transverse sectioning, and frozen in melting isopentane above liquid nitrogen for histological analysis. The rest of the muscle was flash‐frozen in liquid nitrogen for subsequent RNA‐extraction. Approximately 200 mg of the muscle was added to 2 ml MagNA Lyser Green Bead screw‐capped tubes, prefilled with 1.4 mm (diameter) ceramic beads as supplied (PN:03358941001, Roche, Germany) and 1 ml of Trizol (Thermo Fisher Scientific Inc, Waltham, USA). Samples were homogenized using a MagNA Lyser (Roche Diagnostics, Germany) for 40 s at 6 m/s before being placed back on ice. This was repeated five times with 3 min on ice between each repeat to ensure complete disruption of the muscle sample. RNA was extracted using the standard Tri‐Reagent procedure with chloroform/isopropanol for phase separation and precipitation of RNA, with further washing with 75% ethanol. RNA pellets were resuspended in DEPC‐treated water (Thermo Fisher Scientific Inc, Waltham, USA).

### 2.6 RNA library preparation & sequencing

Libraries were constructed from 100 ng of total RNA with Poly‐A tail enrichment of mRNA using NEBNext® Ultra™ II Directional RNA Library Prep with Sample Purification Beads kit, #E7765S (New England Biolabs, MA, USA) as per manufacturers guidelines, by Bart's and the London Genome Centre at Queen Mary, University of London. The resultant‐barcoded libraries were sequenced on an Illumina NextSeq 550 using paired‐end sequencing of 150 bp. Over 12 million reads were achieved per sample. QA/QC data are available in Supplementary File 1.

### 2.7 Bioinformatic processing

FastQ files were imported to Partek® Flow® Genomic Analysis Software Partek Inc. (Missouri, USA) for pipeline processing. Pre‐alignment QA/QC was performed on all reads prior to read trimming below a Phred quality score of 25 and adapter trimming. STAR alignment 4.4.1d was used to align reads to the Rattus Norvegicus, Rnor_6.0 genome assembly.^25^ Aligned reads were then quantified to the Ensembl transcriptome annotation model associated with Rattus Norvegicus Rnor 6.0, release 99_v2. Transcript expression was normalized using DESeq2 median of ratios^26^ and DEGs identified through the Partek® Flow® DESeq2's binomial generalized linear model with Wald testing for significance between conditions set at *p* < .01. Data are presented as fold change ± confidence intervals or log^2^ fold change ± confidence intervals.

### 2.8 Functional analysis & network visualization

Biological interpretation of filtered/selected gene lists was performed in Partek® Flow® Genomic Analysis Software to identify associated KEGG pathways.^27^ To visualize groups of genes with similar temporal changes in gene expression across the time‐course studied, we implemented Self Organizing Map (SOM) profiling of the change in mean gene expression within each condition using Partek Genomics Suite V.7.0 (Partek Inc. Missouri, USA). For normalization, mean Deseq2 values for each group were produced for each gene. The mean of all groups was then shifted to 0 and scaled to 1 standard deviation. SOM plots are presented as normalized expression values (group – mean of all groups). DAVID functional annotation analysis was performed on each SOM and is provided in supplemental file 2. Venn Diagram Analysis was performed using Partek® Flow® and using the VIB/UGent Venn online tool, http://bioinformatics.psb.ugent.be/webtools/Venn/. Publicly available data were taken for comparison from the following studies.^7^, ^8^, ^11^, ^17^, ^19^ HOMER (v4.11) motif analysis was performed on DEGs at each training timepoint, to identify enrichment of known motifs (6–12 bp long) in the gene body and up to 2 kb upstream of the transcription start site. GeneMANIA multiple association network analysis^28^ was performed in Cytoscape 3.8.2 and interaction networks were selected based on physical protein interactions, shared pathways, and shared protein domains.

### 2.9 Immunohistochemistry and MyoVision 2.0 analysis

Histological samples were sectioned at 10 μm from the mid‐belly of the TA and labeled with 2 antibody mixtures to assess fiber size and myonuclear content in all four fiber types. Solution 1 consisted of antibodies against dystrophin (#PA5‐32388 Thermofisher Scientific) (1:200), type IIB myosin (DSHB: BF‐F3) (1:100), and type IIA myosin (DSHB: SC‐71) (1:100) in immunobuffer (IB): PBS (10 mM phosphate pH 7.4, 150 mM NaCl), 50 mM glycine (Merck 1.02401_1000), 0.25% BSA, 0.03% saponin (Sigma S‐7900), 0.05% sodium azide. Solution 2 consisted of antibodies against dystrophin (#PA5‐32388 Thermofisher Scientific) (1:200), type IIX myosin (DSHB: 6H1) (1:100), and type I myosin (DSHB: BA‐D5) (1:100) in IB and were all later counterstained with DAPI for myonuclear identification. Images were captured under 20x magnification using a widefield fluorescent microscope (Leica DMB 6000, Wetzlar, Germany). Multiple images of the whole TA cross section were automatically stitched together using the tilescan feature in the Leica Application Suite and transferred to the MyoVision 2.0 program for myofiber detection and subsequent morphological and fiber‐type‐specific characterization as described previously.^24^

### 2.10 Statistical information

Muscle mass data are presented as the % change between the left experimental hind‐limb and right internal contralateral control hind‐limb for overall muscle mass (mg/kg bodyweight), fiber CSA, that is, the absolute difference expressed as a percentage of the control limb value. The resultant percentage changes were then compared via one‐way ANOVA, followed by Tukey's post hoc analysis to confirm differences between groups. For body mass and fiber type‐specific analysis, absolute values of fiber type proportion, fiber CSA, were compared between groups using one‐way ANOVAs, followed by Bonferroni post hoc analysis to confirm differences between groups. Significance was set at *p* < .05 for all morphological statistical analyses, performed in GraphPad Prism 9.0 software. All data are presented as mean ± standard deviation (SD).

## 3 RESULTS

### 3.1 Daily training leads to progressive hypertrophy, increased mitochondrial content, and changes in myosin heavy chain isoforms

**FIGURE 1:**
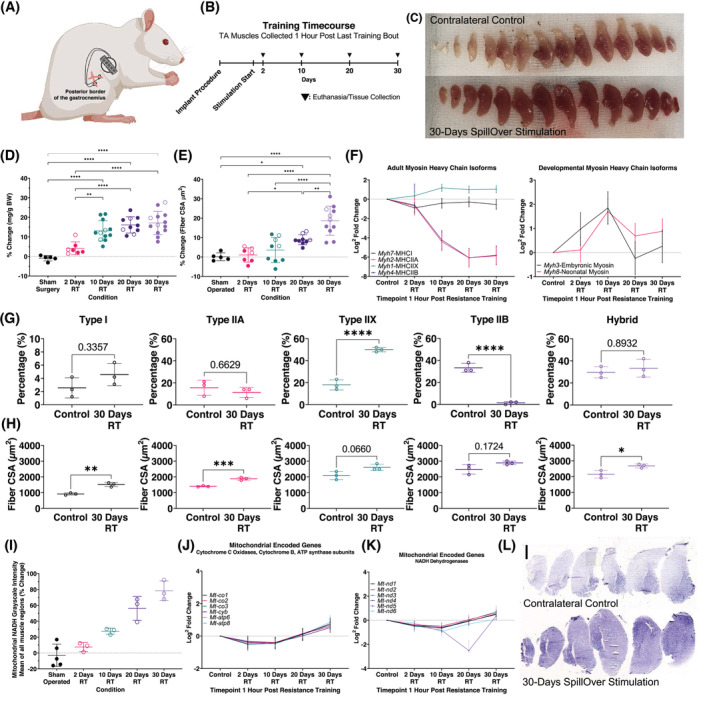
Morphological, mitochondrial, and MyHC isoform adaptations to resistance training. (A) An IPG within the peritoneal space supplied electrodes passed subcutaneously to the left hindlimb. The anode under the tibial nerve and the cathode under the CPN produced supramaximal activation of the dorsiflexors including the tibialis anterior muscle, with co‐contraction of the plantarflexors to provide appropriate resistance. (B) Timecourse of training and daily training pattern. (C) Representative image of transverse tibialis anterior muscle slices, top (right limb, contralateral control), bottom (left limb, 30 days of training). Left to right (distal to proximal). (D) Percentage changes in muscle mass between trained and untrained contralateral control TA muscles in mg/g/body weight across timecourse of training with Tukey post‐hoc analysis. (E) Average percentage changes in fiber cross‐sectional area between trained and untrained contralateral control TA muscles with Tukey post‐hoc analysis. (F) Log^2^ fold changes in adult and developmental myosin heavy chain isoform mRNA. (G) Percentage changes in myosin heavy chain isoforms, assessed by immunohistochemistry. (H) Percentage changes in fiber type‐specific cross‐sectional area. (I) Percentage changes in mitochondrial NADH‐TR staining averaged across all muscle regions (12 cross sections per muscle) between left stimulated and the right unstimulated contralateral control tibialis anterior muscles across the timecourse of training with Tukey post hoc analysis. (J) Log^2^ fold changes in mtDNA encoded gene expression. (K) Log^2^ fold changes in mtDNA encoded NADH dehydrogenase gene expression. (L) Representative image of whole TA cross‐sections stained with NADH‐TR, top (contralateral control), bottom (30 days of training). Left to right (distal to proximal). The black scale bar indicates 2 mm. Morphological Data: *p ≤ .05. **p ≤ .01. ***p ≤ .001. ****p ≤ .0001. Mean ± standard deviation. mRNA Data: log^2^ fold changes ± confidence intervals from DeSeq2 analysis available in Supplementary File 1.

As previously reported in a separate study,^24^ daily SpillOver training results in a progressive increase in muscle mass after 2 (4.2 ± 3.3%, *p* = .37), 10 (13.1 ± 5.2%, *p* < .0001), 20 (16.1 ± 4.2%, *p* < .0001), and 30 days (17.1 ± 5.9%, *p* < .0001), compared with sham surgery (−0.9 ± 1.4%). The increase in muscle mass plateaus between 10 and 30 days of training (*p* = .23), (Figure 1D). Fiber cross‐sectional area (CSA) progressively increases after 2 (1.1 ± 3.7%, *p* = .99), 10 (3.6 ± 6.4%, *p* = .77), 20 (8.8 ± 2.6%, *p* = .04), and 30 days (18.7 ± 7.5%, *p* < .0001), compared with sham surgery (0.1 ± 2.1%), (Figure 1E).

The fiber type distribution after 30 days of training was assessed immunohistochemically with the average cross‐sectional area of each fiber type (Figure 1G,H). Figure 1F shows the mRNA abundance of adult and developmental myosin heavy chain isoform transcripts (*Myh1‐4, 7, and 8*) via RNA‐sequencing across the time course of training. The main change was the increase in type IIX fibers after 30 days of training (18 ± 4.59% vs. 50 ± 4.1%, *p* < .0001), with a decline in the proportion of type IIB fibers (33.33 ± 4.04% vs. 1.4 ± 1.22%, *p* < .0001) (Figure 1G). At the mRNA level, type IIX myosin (*Myh1*) expression increased after 10, 20, and 30 days, versus control levels (~1 log^2^ fold change, *q* < .01), with no difference between each training time point. In line with our immunohistochemical analysis, MyHCIIB (*Myh4*) gene expression remained unaltered after 2 days of training (−0.61 log^2^ FC, *p* = .21) but then declined sharply after 10 (−4.22 log^2^ FC, *q* < .0001), 20 (−6.07 log^2^ FC, *q* < .0001) and 30 days of training (−5.81 log^2^ FC, *q* < .0001). There was no difference in this transcript between 20 and 30 days suggesting that downregulation of this transcript had plateaued at a level of ~5000 reads per million, (Figure 1F). Such selective regulation of myosin isoform transcripts, especially between the 2B and 2X isoforms is remarkable. MyHCIIA gene expression (*Myh2*) decreased progressively in relation to control muscles after 2 (−0.71 log^2^ FC, *p* = .14), 10 (−4.41 log^2^ FC, *q* < .0001), 20 (−6.08 log^2^ FC, *q* < .0001), and 30 days training (−5.87 log^2^ FC, *q* < .0001), respectively. Figure 1F shows similar patterns for MyHCIIA (*Myh2*) and MyHCIIB myosin (*Myh4*) expression, suggesting a coordinated regulatory mechanism.

Embryonic (*Myh3*) and neonatal myosin (*Myh8*) expression remained low at all time points (less than 10 reads per million) despite a small increase in embryonic myosin after 2 (*q* < .001) and 10 days of training (*q* < .0001), before returning to control levels. As expected of the myogenic program, increases in neonatal myosin heavy chain occurred after embryonic expression, reaching significance after 10 (*q* < .001), 20 (*q* < .001), and 30 days (*q* = .001). Despite significant fold changes, the mRNA reads for these genes were less than 10 per million and so probably have little influence on the function: this highlights the absence of a degeneration/regeneration cycle in this model, as histological assessment showed previously.^24^

Fiber type‐specific measurements of fiber area revealed that all fiber types hypertrophied in response to the 30 days of training, type I (918.8 ± 69.7 μm^2^ vs. 1514 ± 134 μm^2^ (~65%), *p* = .0022), type IIA (1405 ± 44.9 μm^2^ vs. 1883 ± 92.6 μm^2^ (~34%), *p* = .0008), type IIX (2023 ± 171.6 μm^2^ vs. 2611 ± 201.3 μm^2^ (~29%), *p* = .0199), type IIB, (2476 ± 298 μm^2^ vs. 2888 ± 115.8 μm^2^ (~17%), *p* = .018) and hybrid fibers (2154 ± 237.6 μm^2^ vs. 2684 ± 124.9 μm^2^ (~25%), *p* = .024). The percentage change was highest in the smallest type I fibers (~65% increase), by contrast with fast fibers (21–34% increase), Figure 1H. With the almost complete shift from IIB (*Myh4*) to IIX (*Myh2*) myosin heavy chain isoforms as assessed through immunohistochemistry and RNA‐sequencing, it may be that IIX fibers did not hypertrophy to as great an extent as reported above because some naturally larger type IIB fibers had transformed to become part of the IIX population. Control IIB fiber CSA (2476 ± 205.2 μm^2^) was only slightly lower than trained IIX fibers (2611 ± 201.3 μm^2^, *p* = .006), resulting in a 9.8% increase, rather than a 29% increase in fiber CSA for type IIX, suggesting that in this model in which all fibers are activated equally and simultaneously, slower fibers show the greatest hypertrophy.

NADH‐TR grayscale intensity representing mitochondrial enzyme activity across all muscle regions progressively increased after 2 (7.5 ± 5.7%, *p* = .85), 10 (27.4 ± 3.8%, *p* = .03), 20 (56.1 ± 15.1%, *p* < .0001), and 30 days (78.2 ± 12.4%, *p* < .0001), compared with sham surgery (−2.9 ± 14.1%), with no difference between 20 and 30 days (*p* = .2891), (Figure 1I,L). The near linear increase in NADH staining did not, however, correspond to changes in mRNA levels for mitochondrial encoded Complex proteins, Mitochondrial Encoded Cytochrome C Oxidase I, II, and III (*Mt‐co1*, *Mt‐co2*, *Mt‐co3*) cytochrome b (*Mt‐cyb*), ATP Synthase Membrane Subunit 6 (*Mt‐atp6*), ATP Synthase Membrane Subunit 8 (*Mt‐atp8*) and mitochondrial encoded NADH: Ubiquinone Oxidoreductase core subunits 1–6 (*Mt‐nd1‐6*), Figure 1J,K. *Mt‐co1‐3*, *Mt‐cyb*, and *Mt‐atp8* were downregulated (*q* < .05) after 2 days by a log^2^ fold change between −0.31 and − 0.7. At 10 days, *Mt‐co1‐3*, *Mt‐cyb*, and NADH dehydrogenase subunits 1–6 (*Mt‐nd1‐6*) were downregulated versus control (*q* < .05), with a log^2^ fold change between −0.39 and − 0.93. After 20 days, expression returned to control in all mitochondrial encoded genes except NADH dehydrogenase subunit 5 which was significantly lower than at all other time points (−2.51 log^2^ fold change, *q* < .05). Significant upregulation of any of these mitochondrial genes 1‐h post‐exercise only occurred after 30 days of training (*q* < .001), consisting of 0.5–0.75 log^2^ fold changes, except for NADH dehydrogenase subunits 5 and 6 that did not differ from control levels, (Figure 1J,K). It is possible that the early increases in the oxidative capacity implied by increased NADH‐TR staining may be regulated by increased protein stability and a reduction in proteolysis of mitochondrial components.^29^

### 3.2 Self‐organizing mapping reveals five distinct temporal clusters of gene expression during hypertrophy

**FIGURE 2:**
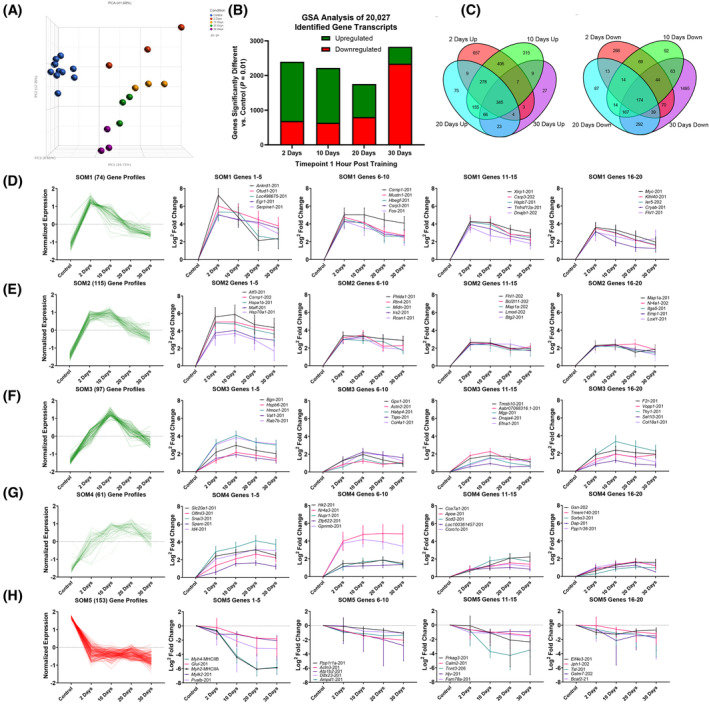
Clustering of the patterns of change in gene expression with daily resistance exercise. (A) Principal components analysis (PCA) of control (blue), 2 days of resistance training (red), 10 days of resistance training (yellow), 20 days of resistance training (green), 30 days of resistance training (purple). PC1 accounted for 19.7% of the variance in the data, PC2 12.4% and PC3, 9.6%, respectively. (B) 20027 different gene transcripts were identified across all samples, and these were filtered following differential gene set analysis between the control group (*n* = 12) and each SpillOver resistance training group (*n* = 3) at each timepoint. (C) DEGs that are common among comparisons. (D–H) The top 500 DEGs across all timepoints from (B), formed five distinct clusters following self‐organizing mapping (SOM) temporal analysis illustrated by the mean (green line plot) and individual data points (gray) for control, 2, 10, 20, and 30 days of SpillOver training. Division into five clusters was based on the inflection point of the minimum centroid distance between clusters, that is, where differences between a larger number of clusters become negligible. SOM1 = 74, SOM2 = 115, SOM3 = 97, SOM4 = 66, SOM5 = 174). The top 20 genes, by order of significance are plotted for each SOM across our timecourse of resistance training. Data are presented as log^2^ fold changes ± confidence intervals.

Principal component analysis (PCA) on all identified gene transcripts showed clustering of gene transcript responses with respect to training and the length of training (Figure 2A). All contralateral control samples clustered together independent of the length of training in the contralateral, unexercised limb. Acute responses after 2 and 10 days of training were similar, but there is distinct separation between 2 and 10 days versus 20 and 30 days, and further division between 20 and 30 days across PC1 (66.43%), (Figure 2A). DeSeq2 normalization and differential expression analysis identified DEGs with a cut off at (*q* < .05) across the timecourse of adaptation. Figure 2B shows that, from the 20 027 gene transcripts identified across all tibialis anterior samples, 2398 were differentially expressed at the 2‐day timepoint, and 71.3% were upregulated. A similar pattern was present after 10 days, with 71.3% of 2218 DEGs upregulated. After 20 days, the number of DEGs decreased to 1755, and only 54.4% were upregulated. After 30 days of training, acute exercise upregulated only 484 genes (17.1%) and downregulated 2828 (82.9%) genes (Figure 2B). Across all timepoints, we identified 519 DEGs that were always responsive to exercise, 66.5% of which were upregulated, Figure 2C.

We performed unsupervised hierarchical clustering of the top 500 DEGs across the timecourse versus control samples and found good similarity of fold changes between samples from the same timepoint, and clear temporal trajectories of expression in the DEGs between 2, 10, 20, and 30 days that was not observed in control samples (Figure 2D–H). SOM temporal analysis of these 500 genes produced five main temporal patterns or clusters containing 74, 115, 97, 61, and 153 genes, respectively. A full list of DEGs at each timepoint (Figure 2B), Venn diagram analysis (Figure 2C), and genes identified by SOM cluster analysis with functional enrichment analysis (Figure 2D–H) can be found in Supplementary Files 1 and 2.

### 3.3 Pathway enrichment of training status‐dependent and training status‐independent responses after a bout of resistance exercise

**FIGURE 3:**
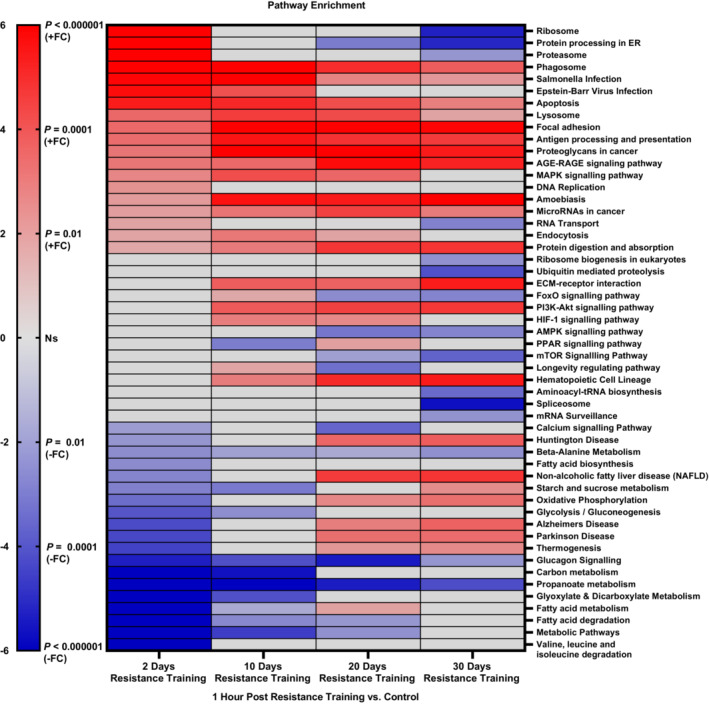
Pathway enrichment of training status‐dependent and training status‐independent responses to acute resistance exercise. KEGG pathway enrichment of DEGs following acute resistance exercise ordered by significance and fold change after 2 days of resistance training.

Pathway enrichment analysis was performed on DEGs at each timepoint and then ordered by fold change and significance, Figure 3. A full list of significantly altered pathways is in Supplementary File 2. Genes associated with “ribosome,” “proteasome,” and “protein processing in endoplasmic reticulum” were upregulated 1‐h post‐exercise after just 2 days of training, probably reflecting the early requirement for increased ribosomal content, translational capacity, and protein synthesis.^30^, ^31^, ^32^ The latter two pathways with “ribosome biogenesis in eukaryotes” are downregulated 1‐h post‐exercise after 20 and 30 days of training which may indicate the reduced stimulus for growth. There is consistent upregulation of “Epstein Barr virus infection,” “salmonella infection,” “phagosome,” “antigen processing and presentation,” “amoebiasis” and “lysosome,” indicative of the immune/inflammatory response previously reported after exercise.^8^, ^33^ Alongside these inflammatory response pathways, “focal adhesion” was always transcriptionally responsive to exercise, independent of training status, and has been well characterized in terms of muscle adaptation, costamerogenesis, load‐activated phosphorylation cascades (integrin signaling), muscle stiffness, and regulation of insulin sensitivity.^34^, ^35^

**FIGURE 4:**
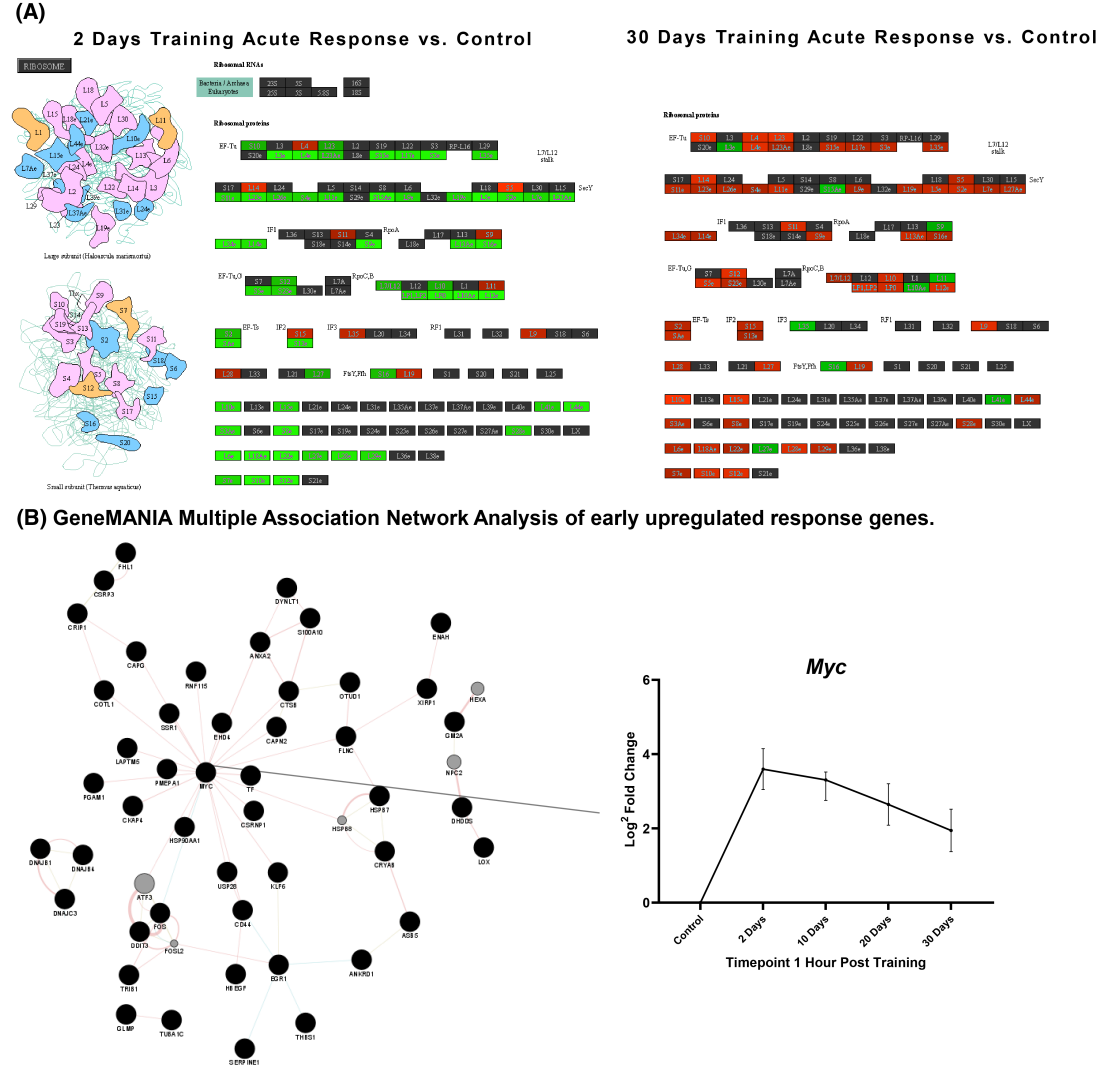
*Myc* is implicated as a central regulator of the early hypertrophic response with GeneMANIA network analyses. (A) Changes in expression of genes associated with the KEGG term, “Ribosome,” 1 h after acute exercise on the 2nd day of training shows a large upregulation (green) of genes relative to control muscles. (“Ribosome” is the most significant pathway at this timepoint). By 30 days, despite ongoing resistance training, genes associated with “Ribosome” are significantly downregulated 1 h after the exercise session. (B) GeneMANIA multiple association network analysis of the early response genes identifies *Myc* Proto‐Oncogene, bHLH TF as the master regulator of the response. *Myc* appears in SOM cluster 1 and its expression level is progressively less elevated in response to acute resistance exercise, as muscle becomes more trained.

All pathways associated with metabolism are downregulated in the exercise naïve muscle acutely after exercise and in most cases, this downregulation remains after 10 days of training. As the muscle mass plateaus between 20 and 30 days of training, however, there is a change in the acute transcriptional response shifting from a “pro‐growth” program with downregulation of pathways relating to metabolism, to positive regulation of the “oxidative phosphorylation” pathway after the acute bout of exercise along with “Parkinson disease,” “Alzheimer's disease,” “Huntington disease,” and “non‐alcoholic fatty acid liver disease” pathways by 30 days of training. In the glucagon signaling pathway, there was a consistent reduction (independent of training status) in the expression of genes relating to glycogen breakdown including glycogen phosphorylase (*Pygm*) and Phosphorylase b kinase (*Phkg1*), which phosphorylate and activate glycogen phosphorylase and therefore promote glycogenolysis. These changes may be secondary to increases in insulin. Figure 4A shows the dramatic shift in the “ribosome” pathway whose early upregulation changes by 30 days to downregulation, so that ribosomal‐associated gene expression is lower in the trained muscle than the untrained, contralateral control muscles. We performed training daily, whereas many resistance exercise studies train animals every 3 days according to the American College of Sports Medicine (ACSM) guidelines for human training.^12^ The lack of recovery between bouts may promote a shift of the transcriptional response from “growth” (achieving about 15% hypertrophy), to “ensuring enzyme and substrate availability” as the training duration increases.

### 3.4 Network analysis implicates *Myc* as a central regulator of hypertrophic adaptation and the ribosome

As SOM1 and SOM2 showed the most similar relationship with the increase and cessation of muscle growth, we selected these clusters of genes for GeneMANIA network analysis. Figure 4B shows the TF *Myc*, as a central regulator of the gene expression response. *Myc* is in SOM1, showing an initial upregulation followed by a gradual decline in its response to acute resistance exercise over 30 days. The involvement of *Myc* in exercise adaptation is well established, reported after synergist ablation in the rat,^36^ and in chickens following mechanical stretch of flight muscles.^37^ Its expression changes in both myonuclei and satellite cells. MYC is a bHLH TF and binds to the non‐canonical E‐box sequence (5’‐CACGTG‐3′), or non‐canonical sites in gene promoters or enhancers that regulate transcription,^38^ following heterodimerization with its transcriptional partner MAX (Myc‐associated factor X). MYC controls regulation of the core molecular clock,^39^ cell cycle, cell growth, glycolysis, oxidative metabolism, glutamine metabolism, mitochondrial biogenesis, ribosomal biogenesis, and RNA polymerase activity.^40^ The availability of target E‐boxes for MYC depends on chromatin accessibility and chromatin modifying co‐factors near to gene enhancer regions that are extremely tissue‐specific,^41^ and unknown in adult skeletal muscle tissue.^42^

Overexpression of *Myc* via adeno‐associated virus transfection into adult skeletal muscle increased DNA polymerase subunit *Pola1* mRNA, *Rn45s* pre‐rRNA, total RNA, and muscle protein synthesis, independent of mTORC1 and mainly regulates ribosome biogenesis.^43^, ^44^, ^45^ Our pathway analysis of early DEGs (Figure 3) shows that “ribosome” is the most significantly upregulated biological process, coinciding with the peak of *Myc* expression at 2d. Temporal analysis of the “ribosome” pathway (Figures 3 and 4A) shows a gradual decline in that upregulation, until negative regulation of “ribosome” after 30 days of training, which coincides with the reduction in amplitude of *Myc* gene expression (Figure 4B).

### 3.5 Transcription factor motif enrichment of training status‐dependent and training status‐independent responses after a bout of resistance exercise

**FIGURE 5:**
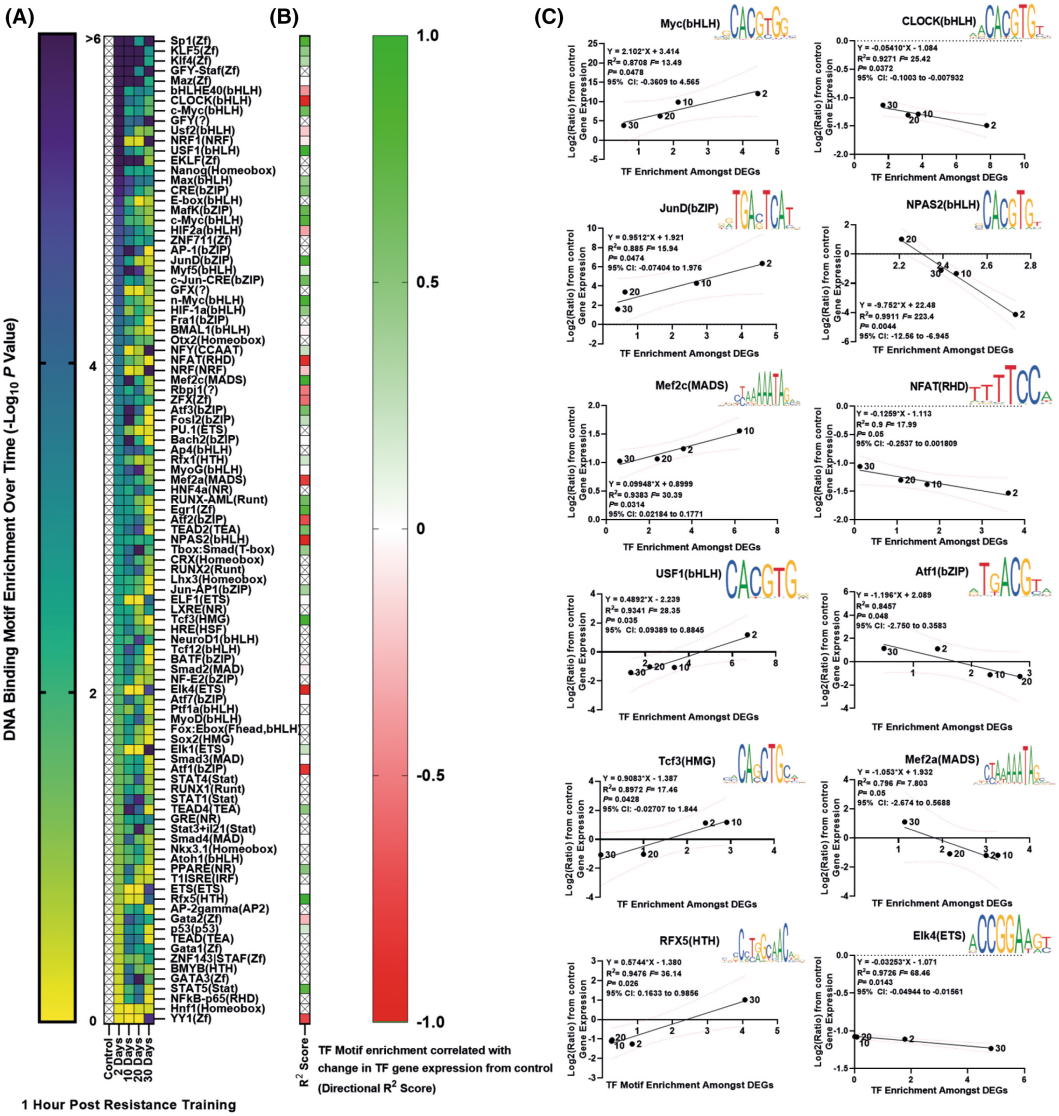
Transcription factor motif enrichment over the timecourse for differentially expressed genes and correlations with the transcription factor gene expression. (A) DEGs from Figure 2B were analyzed for motif enrichment in the gene body and 2 kb upstream of the transcription start site and plotted in a heatmap to show the enrichment over time. (B) TF motif enrichment correlated with change in TF gene expression from control. *R* ^2^ scores are normalized to direction of relationship. X indicates lack of TF gene expression. (C) Top six positive (left column) and top six negative (right column) Pearson's *R* ^2^ correlations, ordered by significance, and associated DNA binding motif.

To gain insight into the TFs that control the DEG (Figure 2B,C) responses according to length of training, we performed hypergeometric optimization of motif enrichment (HOMER) to identify enriched transcription factor motifs in the gene body and up to 2 kb upstream of the transcription start site within DEGs. As many TFs have low mRNA abundance and read depth within our RNA‐seq analysis was moderate, changes in expression of these TF mRNAs may be missed, even though their target genes are still differentially expressed. In fact, of the top 100 TF motifs identified, 63 also had corresponding mRNA values at each timepoint. Pearson's analysis quantified the correlation between gene expression of the TF itself at each timepoint and motif enrichment in the sequence for the DEGs and 2 kb upstream of the DEG. *Myc* gene expression was strongly positively correlated (*R* ^2^ = 0.8708, *p* = .*0478*) with its own DNA binding motif (VCCACGTG) enrichment among the DEGs, suggesting that its increased expression has a direct relationship with the number of differentially expressed target genes. The largest change in *Myc* expression occurred at 2 days which coincided with the largest number of DEGs enriched with the MYC DNA binding motif. The smallest change in gene expression and DNA binding motif enrichment was found after 30 days of training, Figure 5C. Interestingly, MYC's heterodimeric partner MYC‐associated factor X, *Max*, is consistently downregulated in the acute response to SpillOver training. It is almost completely switched off at 20d and 30d with less than 10 reads per million (Supplementary File 1). As mentioned previously, the ribosomal biogenesis response seems to be conserved across species^46^ and decline of *Myc* expression in the acute response to resistance exercise as training status increases has been reported in humans after 6 and 12 weeks of training.^47^, ^48^ The latter study also reported that 45 s‐pre‐RNA followed a similar trend. A similar consensus between species was found in the rDNA methylation within myonuclei following acute hypertrophic stimuli in mice and humans. Methylation patterns were modified in enhancer, intergenic, and non‐canonical regions but the methylation status of the promoter region did not change.^32^ It will be interesting to discover whether post‐translational modifications or changes to the chromatin accessibility of E‐box binding sites, can explain reduced *Myc* and ribosomal‐associated gene response to acute resistance exercise. Both Upstream Transcription Factor 1 (*Usf1*) (SGTCACGTGR), (*R* ^2^ = 0.9341, *p* = .035) and Transcription Factor 3 (*Tcf3*) (ASWTCAAAGG), (*R* ^2^ = 0.8972, *p* = .0428) which also bind E‐boxes, show a similar pattern to *Myc*. Larger increases in gene expression from control in the early training responses lead to higher DNA binding motif enrichment in the DEGs, and a reduction in gene expression in trained muscle was associated with decreased DNA binding motif enrichment among DEGs.

By contrast, E‐box circadian TFs *Clock* (GHCACGTG) (*R* ^2^ = 0.9272, *p* = .0372) and *Npas2* (KCCACGTGAC) (*R* ^2^ = 0.9911, *p* = .0044) are the top two most significantly *negatively* correlated TFs in the HOMER analysis. Both of these mRNAs display the largest decrease in expression from control levels in the early training timepoints (2 and 10 days), but still have elevated DNA binding motif enrichment among DEGs. As these proteins share similar E‐box binding sites with MYC, it is likely that the enrichment of E‐box sequences within the DEGs is a result of MYC activity. Interestingly, the expression of CLOCK's TF complex partner *Arntl*, had no relationship with the enrichment of the ARNTL/BMAL1 binding motif (GNCACGTG) enrichment among DEGs (*R* ^2^ = −0.05, *p* = .98). The larger reductions in *Clock/Npas2* gene expression during the early training responses compared with the later training responses, may be indicative of phase shifts within the muscle molecular clock because exercise was delivered during the start of the rat's inactive phase.^49^ Further analysis of the DEGs revealed that 19.82% of them at 2d, 32.13% at 10d, 21.75% at 20d, and 20.04% at 30d are known to oscillate in a circadian manner in murine skeletal muscle according to the CircaAge database,^50^ (Supplementary File 3), suggesting they are under regulation of the molecular clock.

*Jund* (ATGACGTCATCV), a member of the activator protein 1 (AP‐1) TF complex, also demonstrated significant positive correlation (*R* ^2^ = 0.885, *p* = .0474). TF complexes consisting of FOS, JUN, activating transcription factors (ATFs), and CREB proteins are well known for regulating the acute transcriptional response to biological stress and exercise, binding to (5TGACTCA) sites and cAMP response elements (CRE) (CACGTG) activating downstream signaling cascades to reprogram metabolism.^51^, ^52^ *Mef2c*, a well‐characterized MADS‐box binding TF also shows a positive correlation, with higher gene expression leading to greater DNA binding motif enrichment (DCYAAAAATAGM) among DEGs, and the earlier timepoints showing the greatest increase in gene expression and downstream target genes. MEF2C regulates fiber type, sarcomeric gene expression, and metabolism,^53^ is highly responsive to contractile activity (changes in calcium),^54^ and can bind directly to the PGC‐1 alpha promoter, regulating mitochondrial biogenesis.^55^, ^56^ Interestingly, *Nfatc3* gene expression was reduced at all timepoints versus control muscle, with the largest reductions in gene expression occurring after 2 days of training and the smallest reduction in gene expression occurring after 30 days of training. Our data suggest that during the largest reduction in gene expression of *Nfatc3*, NFAT DNA binding motif enrichment (ATTTT) among DEGs was at its highest. NFAT is well known for promoting fast to slow adaptation in myosin gene expression and fine‐tuning metabolism to fatty acid oxidation,^57^, ^58^ which suggests that in our model, the decreases in gene expression of *Nfatc3* do not associate with the activity of the protein itself.

### 3.6 Comparative transcriptome analysis between species and models reveals only 10 consistently reported regulators of resistance‐exercise‐induced hypertrophy

Despite several recent reports, there is not strong consensus on the transcriptional underpinnings of adaptation to exercise and specifically to resistance exercise. This may be the consequence of large variability in the origin and the activation history of the samples studied so that it is difficult to identify “master regulators,” or key gene interactions that might be targeted to improve exercise responsiveness or monitored to achieve more effective adaptation through individualized, prescribed exercise. Samples themselves may differ in species, maturity, metabolic state, training history, timepoint post‐exercise, exercise type (reps, sets, rest, contraction modality), and muscle group.^21^ There are also differences in the detail and versions of the genome assembly used which may further contribute to differences in identified genes. In rodent studies, researchers often study the “anti‐gravity” ankle plantarflexors, but individual muscles vary considerably within this muscle group. Additionally, there is little consensus on the appropriate normalization methods and filtering used in next‐generation RNA‐sequencing, with different exercise studies adopting completely different approaches leading to differing outcomes.

**FIGURE 6:**
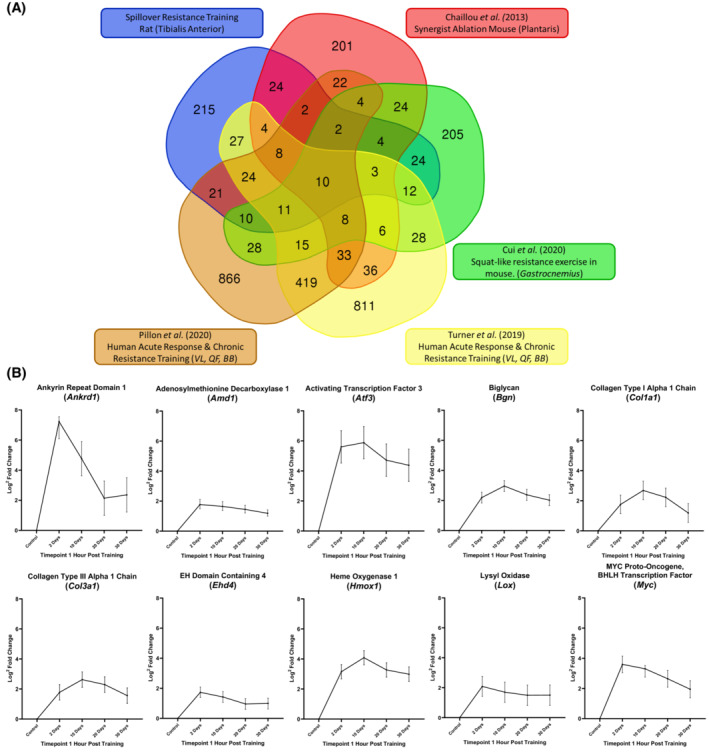
Conserved regulators of hypertrophy across species and models. (A) Venn diagram analysis of the top 400 DEGs ordered by significance in three distinct rodent models of hypertrophy and two meta‐analyses of data from studies of resistance exercise in humans. Human datasets contain both the top 750 genes following acute resistance exercise and top 750 genes associated with chronic resistance exercise in humans from each publication (Turner, Seaborne and Sharples^17^; Pillon, Gabriel, Dollet, Smith, Puig, Botella, Bishop, Krook and Zierath^19^). (B) The timecourse of log^2^ fold changes ± confidence intervals for the 10 genes found to overlap between all transcriptomic datasets. Full gene lists across the Venn analysis are available in Supplementary File 4.

There is no prior dataset showing changes in gene expression over a similarly well‐controlled timecourse of muscle growth as we present here, and we hope that the availability of this dataset will stimulate further analyses. However, we made comparison with five existing datasets from human and rodent exercise experiments.^7^, ^11^, ^17^, ^19^ By overlapping the top 400 genes from Chaillou, Lee, England, Esser, and McCarthy^7^ and Cui, Drake, Wilson, Shute, Lewellen, Zhang, Zhao, Sabik, Onengut, and Berr^11^ (by order of significance) and the top 750 DEGs noted after either acute or long‐term exercise from two meta‐dataset analyses (Pillon, Gabriel, Dollet, Smith, Puig, Botella, Bishop, Krook, and Zierath^19^ and Turner, Seaborne and Sharples^17^) with our SpillOver resistance training dataset, we hoped to identify DEGs that might be specific to individual rodent models of exercise, genes that were shared across rodent models, and overlap with DEGs identified in meta‐analyses of the acute phase following human resistance exercise and in resting, but, chronically trained muscle, Figure 6A.

There is surprisingly little correspondence between the two systematic meta‐analysess^19^ and Sharples groups,^17^ even when their lists of most significant genes are extended to include both acute and chronic resistance training responses and the stringency is lowered to include 750 genes from each dataset. When overlapped, only 528 of 1500 genes (35%) were noted in both human resistance training meta‐analyses, presumably due to differences in datasets studied and statistical approaches used. Furthermore, 419 genes (79%) of those 528 genes were not represented in the top 400 most significantly DEGs in the three rodent models of resistance exercise, perhaps highlighting a group of genes that are typically responsive to exercise in humans, but not in rodents, which requires further investigation. Some of these differences may arise from differences in coverage and annotations of genes between mouse, rat, and human genomes, and emphasizes the need for improvement in the bioinformatic information available for such comparisons.

We then compared the most significant DEGs in our SpillOver resistance training dataset with both human datasets and 134/400 genes (33.5%) overlapped. Thirty‐five of these (8.8%) were specific to the overlap between SpillOver training and the Turner, Seaborne and Sharples^17^ human resistance training dataset and 46 genes (11.5%) were specific to the overlap between SpillOver training and the Pillon, Gabriel, Dollet, Smith, Puig, Botella, Bishop, Krook, and Zierath^19^ human resistance training dataset. A further 53 (13.3%) were found to overlap between SpillOver training and both human resistance training meta‐analyses.

Furthermore, the changes in gene expression associated with the hypertrophy induced by synergist ablation overload are distinct from the changes we have observed. Only 57 genes (14.3%) overlapped with SpillOver training, 24 of which were noted only in synergist ablation in mice and SpillOver training in rats. There was slightly more overlap observed between SpillOver training and voluntary weightlifting in mice (66 genes, 16.5%), of which 24 were found only with SpillOver training and voluntary weightlifting. There was similar overlap observed between synergist ablation and voluntary weightlifting in mice (61 genes, 15.25%), of which 24 genes were unique between these models. This analysis emphasizes that the synergist ablation model, while it produces eventual hypertrophy, is a model of continuous overload to failure with extensive damage/regeneration, rather than a model of training‐induced muscle growth. This is an important caveat because the changes in gene expression with synergist ablation are often taken as representative of muscle growth in response to strength training. The acute response that we capture with our timepoints may also not be directly comparable with the voluntary squat‐like weightlifting model that provided muscle transcriptome analysis at a single timepoint between resting and 8‐week trained gastrocnemius muscle.^11^ There were 19 genes (4.5%) from the top 400 SpillOver genes that appeared in all three rodent models, four of which (1% of the 400 SpillOver genes) were only found in rodent models and did not appear in any of the human datasets. Those genes were Complement C1q B Chain (*C1qb*), Colony stimulating factor 1 receptor (Csf1r), Serpin Family E Member 1 (*Serpine1*), Microfibril‐associated protein 4 (*Mfap4*), appearing in SOM2, 2, 1, and 2, respectively. They have previously been associated with macrophages,^59^, ^60^ inflammation, and extracellular matrix remodeling,^61^ suggesting that these transcriptional responses may be more prominent in rodents following exercise.

Of the top 400 genes significantly altered after SpillOver training, 215 (53.8%) were specific to SpillOver training. These responses may be specific to rats following exercise and require further investigation. Only 10 genes were found to overlap across all models of resistance exercise and species (2.5% of the 400 differentially expressed SpillOver genes) including our main hub gene *Myc* (SOM1), identified through GeneMANIA analysis, Figure 6A,B. The others were Adenosylmethionine decarboxylase 1 (*Amd1*) (SOM5), Ankyrin repeat domain 1 (*Ankrd1*) (SOM1), Activating Transcription Factor 3 (*Atf3*) (SOM2), Biglycan (*Bgn*) (SOM3), Collagen Type I Alpha 1 Chain (*Col1a1*) (SOM3), Collagen Type III Alpha 1 Chain (Col3a1) (SOM3), EH Domain Containing 4 (Ehd4) (SOM1), Lysyl Oxidase (*Lox*) (SOM2), and Heme Oxygenase 1 (Hmox1) (Figure 6B). These 10 genes all show a rise in expression with training, followed by a return to control levels over the course of our 30‐day study. We also highlight other genes that were present in all but one dataset that we compared in our Venn diagram analysis, which may also be important factors in the hypertrophic process/muscle adaptation (Supplementary File 4).

## 4 DISCUSSION

Resistance exercise promotes increases in muscle size, quality, and strength, which are key predictors for all‐cause mortality across the lifespan.^62^ Transcriptional changes following resistance exercise are required to rewire metabolism and pro‐growth pathways. However, little is known about how the record of previous exercise, or training status, may modulate the amplitude or temporal pattern of the transcriptional response, which mediates muscle adaptation. Defining and understanding these responses may help us to personalize training regimens to individuals based on their training history. Our study provides a detailed insight into these adaptations in a highly controlled timecourse that is otherwise absent from the current literature. In this study, the interplay between muscle adaptation and the altered gene expression that drives it was explored. Programmed daily exercise causes rapid changes in gene expression that result in physiological modifications in the muscle phenotype after just a few days. These changes in turn modify the acute transcriptional response to exercise. The acute transcriptional response to SpillOver training is strikingly different dependent on training status, and only 345 genes are always upregulated, and 174 genes are always downregulated 1‐h post‐training.

Our model of daily training produces robust hypertrophy, but we also see changes in mitochondrial content and myosin heavy chain isoform expression that are typical of changes associated with endurance training. These changes show that in rat, as also recommended for human, a longer recovery period than 24 h may be required to achieve enhancement of muscle size without loss of speed. They also suggest that the rat is an appropriate model in which to investigate the cellular mechanisms underlying exercise periodization. Changes in most of the mitochondrial encoded genes and in the myosin heavy chain genes do not follow the main patterns identified by our self‐organizing mapping, (Figure 2). Surprisingly, only 50 high‐frequency contractions over a period of 20 min, delivered daily, was enough to elicit a complete silencing of *Myh4* mRNA and the related MyHIIB protein expression and a shift to a muscle predominantly expressing the MyHIIX protein isoform, (Figure 1). We highlight that mitochondrial‐encoded genes show little change in gene expression acutely after exercise, despite progressive increases in mitochondrial content, which may point to the greater importance of translational capacity and RNA stability. Pathways related to ribosomal density, translational capacity, and efficiency are the first pathways to be upregulated in exercise naïve muscle, (Figure 3) in order to elicit growth. These genes are subsequently downregulated as daily exercise continues and as the transcriptome shifts to favor oxidative metabolism post‐exercise.

We identify a number of genes from SOM1‐3 whose responsiveness to exercise appear to correlate with the increase and then stabilization of muscle mass across our timecourse, (Figure 2). Such signals that are activated by unaccustomed exercise and reduced with progressive adaptation may be useful biomarkers of effectiveness in prescribed programs of exercise intended to improve the quantity or quality of muscle. Furthermore, if the reduction in these gene signatures represents an effective adaptation to a particular level of exercise, then that reduction might be used as an indicator that progression to a new level is appropriate. Inappropriate progression can induce muscle soreness or damage and be counterproductive both for athletes and for amateur participants in resistance exercise.

As well as highlighting patterns in the amplitude of gene expression dependent on training status, we identified genes whose response does seem to be conserved across species and models of resistance exercise/mechanical overload, (Figure 6), the majority of which have not been characterized in terms of their role in muscular adaptation to exercise. Of interest, *Ehd4* has been implicated and reported numerous times following resistance exercise,^7^, ^8^, ^18^ yet there is very little discussion of its role in adapting skeletal muscle. *Ehd4* is consistently upregulated in both the proteome and transcriptome following denervation‐induced atrophy and nerve‐silencing‐induced atrophy.^63^ To our best knowledge, the function of *Ehd4* has not been studied in mammalian adult skeletal muscle, but the other Eps15 homology domain‐containing proteins (EHDs) 1–3 have. *Ehd1* and *Ehd2* are implicated in membrane fusion and membrane repair during development and in dystrophic disease. Depletion of either or both, results in decreased fusion efficiency in C2C12 myoblasts^64^ also observed in myoblasts isolated from Ehd1‐null mice, concomitant with reduced fiber size.^65^ In those smaller fibers there was decreased vesicle trafficking and overgrown transverse t‐tubules.^65^ *Ehd3* is upregulated in human and mouse heart tissue after injury, suggesting a role in tissue repair.^66^ *Ehd4* helps with transport of protein from the early endosome to the endosome recycling center in the infarcted heart.^67^, ^68^ In *Torpedo Californica*, EHD4 is localized to perinuclear regions and primary synaptic clefts.^69^ Single nuclei RNA‐seq (snRNA‐seq) from murine TA muscle across the lifespan shows that Ehd4 is enriched in the neuromuscular junction myonuclei^70^ and warrants further investigation. We also identified *Ankrd1* as a conserved upregulated transcription factor across species which was recently highlighted as the most upregulated gene in a myonuclear‐specific transcriptome following synergist ablation.^71^ This gene transcript is enriched in myotendinous junction myonuclei^70^ and as a transcription factor commonly found in both cardiac and skeletal muscle, its transcriptional role in remodeling these tissues and specific muscle regions warrants further investigation.

We also highlight that *Myc* bHLH TF appears to play a critical role in the hypertrophic process and may act as a master regulator of the transcriptional response to resistance exercise, appearing in three separate lines of our investigation. First, it appeared in the top 20 DEGs in SOM1, the temporal cluster that shows genes most responsive in untrained muscle, before decreasing at each timepoint as training status increases (Figure 2D). A similar observation has been made in human muscle in which *MYC* gene expression was acutely elevated after 0, 2, and 4 weeks of training but no longer upregulated in response to acute exercise after 6 weeks.^48^ Other work in humans has shown that untrained individuals have a much higher expression of *MYC* after an acute resistance exercise bout than those who had 6 weeks of training.^47^ This suggests that acute *MYC* expression is reduced with ongoing training even when the absolute load is increased and that the timecourse of 4 weeks of training in rats is comparable to a 6‐week training protocol in humans. Secondly, through GeneMANIA multiple association network analysis (Figure 4), *Myc* appeared as a central hub regulating the genes in the early upregulated response to exercise, which we suggest represent collectively the signal for muscle hypertrophy. HOMER analysis found that there was a strong enrichment of MYC DNA binding motifs in the DEGs, which became less enriched at the later timepoints as training status increased and the muscle stopped growing. This effect was significantly positively correlated with the decreased gene expression of *Myc* (Figure 5C). Lastly, a comparison of five studies, which included three species, three models of mechanical load‐induced hypertrophy, and two large human meta‐analyses of previously published transcriptomic datasets on resistance exercise found that *Myc* was one of only 10 genes that were differentially regulated in all the studies. This suggests that *Myc* plays an important role in the transcriptional regulation of hypertrophy induced by increased mechanical loading. Recent work from Murach et al.^71^ identified *Myc* as a myonuclei‐enriched hypertrophic gene. Inducible skeletal muscle‐specific overexpression of *Myc* identified a number of *Myc* targets including those involved in translation initiation, metabolism of RNA, ribosome, spliceosome, aminoacyl‐tRNA biosynthesis. Of note, a reduction in expression of core circadian clock factors nuclear receptor subfamily 1 group D member 1 and 2 (*Nr1d1*, *Nr1d2*) were shown to be affected by *Myc* overexpression. MYC's function in skeletal muscle and the gradual decrease in amplitude of its gene expression and target DEGs following exercise training should be further explored, including whether epigenetic factors controlling the chromatin landscape and the accessibility of MYC target gene enhancers/promoters may regulate training status‐specific transcriptional responses to exercise training. Understanding changes in transcription factor targets with training status may be important for designing exercise training interventions and therapeutics that maximize growth and muscle health.

## AUTHOR CONTRIBUTIONS

Mark R. Viggars, Hazel Sutherland, and Jonathan C. Jarvis designed experiments. Jonathan C. Jarvis, Hermann Lanmüller, Martin Schmoll, and Manfred Bijak developed the stimulators. Mark R. Viggars, Hazel Sutherland, and Jonathan C. Jarvis performed experiments. Mark R. Viggars and Jonathan C. Jarvis analyzed the data and contributed to interpretations. Mark R. Viggars wrote the manuscript with assistance from all authors. All authors contributed to the research of the published work and have read and approved the final manuscript.

## DISCLOSURES

The authors declare no conflicts of interest.

## Supporting information


Appendix S1

Appendix S2

Appendix S3

Appendix S4

## Data Availability

Raw RNA‐sequencing data (FASTq) are available through GEO Accession, GSE196147. Processed data are provided. RNA‐seq data are available in Supplemental File 1. The data that support the other findings of this study are available in the methods and/or Supplementary Material of this article.
